# Longitudinal ^123^I‐FP‐CIT SPECT Analysis Using DaTQUANT Software in Autopsy Confirmed Dementia with Lewy Bodies Patients

**DOI:** 10.1002/alz70862_110109

**Published:** 2025-12-23

**Authors:** Toji Miyagawa, Scott A. Przybelski, Cynthia Vernon, Hoon‐Ki Min, Leah K. Forsberg, Julie A. Fields, Stuart J McCarter, Tanis J Ferman, Vijay K. Ramanan, Jonathan Graff‐Radford, David T. Jones, Rodolfo Savica, R. Ross Reichard, Joseph E. Parisi, Aivi T. Nguyen, Dennis W. Dickson, Melissa E. Murray, Gregory S Day, Neill R. Graff‐Radford, Erik K St. Louis, Manoj K Jain, Kejal Kantarci, Val J Lowe, Brad F Boeve

**Affiliations:** ^1^ Mayo Clinic, Rochester, MN USA; ^2^ Mayo Clinic, Jacksonville, FL USA

## Abstract

**Background:**

Dementia with Lewy Bodies (DLB) has been overlooked or misdiagnosed as Alzheimer’s disease (AD) dementia due to its frequent concomitant AD pathology with Lewy Body Disease (LBD) pathology. Although autopsy confirmation has been the gold standard for the precise diagnosis of DLB, time‐course ^123^I‐FP‐CIT SPECT (DaT‐SPECT) findings in autopsy‐confirmed DLB are not well characterized. We previously reported that the DaTQUANT z‐score of the more affected side of the putamen has the best discriminatory power in detecting LBD pathology and nigrostriatal degeneration in dementia patients and demonstrated z‐score of −1 as the cutoff value. We sought to examine how DaT‐SPECT findings change over time in autopsy confirmed LBD patients in preparation for its use in LBD targeted clinical trials.

**Method:**

Eight autopsy confirmed neocortical or limbic LBD patients who underwent two or more DaT‐SPECT were included. Nigrostriatal dopamine transporter (DaT) binding and age‐adjusted z‐scores were analyzed using the DaTQUANT 2.0 software (GE Healthcare).

**Result:**

Six patients (75%) were male. The patients had 2.5±0.7 longitudinal DaT‐SPECT scans. Clinical diagnosis at initial scan was: DLB (75%), mild cognitive impairment (MCI) with REM sleep behavior disorder (RBD) (13%), and isolated RBD (iRBD) (13%). Clinical diagnosis at last scan was: DLB (88%) and iRBD (12%). All patients developed dementia during life. Mean age at initial scan/last scan/autopsy were 71.1±7.0/73.4±6.9/75.1±7.3 years old. Time from initial and last scan to autopsy were 4.0±1.9 and 1.8±1.9 years respectively. Seven patients (88%) had neocortical LBD and one (12%) had limbic LBD. Concomitant AD pathology was observed in four patients (two males (33%), two females (100%)). All patients had DaTQUANT putamen z‐score <‐1.0 at their initial scan including in MCI+RBD (‐1.76) and iRBD (‐1.53) patients. In the DLB patients at their initial scans, five (83%) had DaTQUANT putamen z‐score <‐2.0 and one (17%) had z‐score of ‐1.55. All patients (100%) showed DaTQUANT putamen z‐score <‐2.0 at their last scans.

**Conclusion:**

DaTQUANT using cut‐off putamen z‐score of ‐1 was associated with underlying LBD pathology including at their iRBD and MCI stages. Nigrostriatal DaT binding tends to decline over time with DaTQUANT z‐score <‐2 at their last scans.

